# Determination of Cenozoic Sedimentary Structures Using Integrated Geophysical Surveys: A Case Study in the Hebei Plain, China

**DOI:** 10.3390/s25020486

**Published:** 2025-01-16

**Authors:** Yi Yang, Jie Zhang, Junjie Wu, Pei Li, Xingchun Wang, Qingquan Zhi, Guojiang Hao, Jianhua Li, Xiaohong Deng

**Affiliations:** 1 Laboratory of Geophysical EM Probing Technologies, Ministry of Natural Resources, Dongli, Tianjin 300300, China; yyi@mail.cgs.gov.cn (Y.Y.);; 2 The Institute of Geophysical and Geochemical Exploration, Dongli, Tianjin 300300, China

**Keywords:** thick coverage area, sedimentary layer, gravity, magnetotelluric, seismic

## Abstract

The strong multi-stage tectonic movement caused the northwest of the North China Plain to rise and the southeast to fall. The covering layer in the plain area was several kilometers thick. In addition to expensive drilling, it is difficult to obtain deep geological information through traditional geological exploration. In this study, gravity, magnetotelluric (MT) sounding and shallow seismic methods are used to explore the basement relief and stratigraphic structure of the alluvial proluvial area in front of Taihang Mount in the North China Plain so as to understand the geological structure and sedimentary evolution of the area. The gravity anomaly map reveals the basement uplift, depression shape and faults distribution on the horizontal plane in the whole area. The MT profile reflects the geoelectric characteristics of the three-layer distribution in the Cenozoic. The seismic profile deployed on the Daxing Uplift depicts the structural style of the uplift area. The well-to-seismic calibration establishes the relationship between the lithostratigraphic and the wave impedance interface so that we can accurately obtain the shape and depth of the bedrock surface and further subdivide Cenozoic strata. Finally, we have improved the accuracy of interface inversion by using a variable density model based on density logging parameter statistics to constrain the depth of geological interfaces determined through drilling and multi-geophysical methods. Through the combination of geology and comprehensive geophysics, we have obtained the undulating patterns of Paleogene and Quaternary bottom interfaces, the structural styles of the basement and the distribution of faults in the survey area, which provide strong support for the study of neotectonic movement and sedimentary environment evolution since the Cenozoic. The successful application of this pattern proves that geophysical surveys based on prior geological information are an important supplementary tool for geological research in thick coverage areas.

## 1. Introduction

The North China Plain is located in the east of Taihang Mount uplift and the south of the Yanshan fold belt. During the Cenozoic era, the lithosphere in the east of Taihang Mount developed strong extension and rifting, which was accompanied by mantle upwelling and mantle derived magma eruption. These changes formed a basin ridge-type North China Bohai Bay rift basin [1], followed by sustained thermal subsidence sedimentation, which shaped the current topography of the East North China Plain and offshore plain [2]. Since then, the Paleogene, Taihang Mount and Yanshan area have been uplifted intensively, and the North China Plain has been declining continuously. The basement has been subjected to strong crustal extensional faulting, forming a series of graben basins controlled by NE–NNE digressive normal faults [3] and filled and leveled by huge Cenozoic strata [4]. Stratigraphic and chronological studies show that the Taihang and Yanshan Mountains are mainly uplifted in the Quaternary [5,6]. The process of tectonic movement in this area is also the history of sedimentary formation. The rapid accumulation period of sediments in most sedimentary basins is relative to the rapid uplift and growth stage of the plateau [7]. The structural pattern is the main controlling factor for the formation of sedimentary basins, and it is also the key factor for the development of sedimentary sequences [8]. Meanwhile, the sequence characteristics are the response and record of structural activities, so the sedimentary record of basin filling is an indirect reflection of the process of structural movement [9].

The basement structural framework and Quaternary geological structure are the main contents of the geological survey in the deep coverage area. Revealing these geological elements can provide basic geological background information for resource and environment investigation [10] and help to understand structural and sedimentary evolution [11]. In addition, the structure and lithology of the overburden are closely related to groundwater distribution [12], climate change [13] and geological disasters [14,15]. The undulation pattern of the bedrock surface is the response of tectonic movement, and it also controls the later sedimentation. It is an important basis for the reconstruction of paleogeography and geomorphology, and it has a considerable impact on the seismic intensity [16]. Bonnet et al. [17] suggested that the basement morphology can reflect the process of structure and denudation. Pallero et al. [18] suggested that the geometry of sedimentary basins (thickness and basement relief) is essential to understand the role of uplift and erosion in the formation of mountain ranges as well as the structural history. Geophysical methods play an important role in a Quaternary thick cover geological survey. Bielik et al. [19] used gravity data to determine the structure and composition of the Paleogene basement in the Tulik Basin. Krivochieva and Chouteau [20] used magnetotelluric (MT) and time-domain electromagnetic data to constrain the geometry of aquifers, then discovered continuous basalt flow between volcanoes and sedimentary basins, and finally determined the depth of the bedrock and three overlying layers. Jorgensen et al. [21] used TEM data to map the Quaternary hidden valley and obtained the image describing the shape, nature and deposit structure of the valley evolution. Sridhar et al. [22], based on the obvious resistivity comparison between the sediments and the basement, used the helicopter time-domain electromagnetic survey to delineate the basin range and complete the mapping of underground sediments. In order to obtain more reliable results, comprehensive geophysical exploration is used for comprehensive geological surveys. Jorgensen et al. [23] developed a comprehensive interpretation method to quantitatively describe the structure and lithology of the Quaternary Valley in the coverage area, including transient electromagnetic sounding, reflection seismic exploration and exploration drilling. Brooke and Rosenbaum [24] used two-dimensional seismic, aeromagnetic total magnetic strength and Bouguer gravity data sets, combined with outcrop and oil well data, to obtain the structure of the Oroklin sedimentary basin in Texas, Australia. Tschirhart and Pehrsson [25] used the sparse seismic data in the Theron basin and the Precambrian geological data near the basin to delineate the basin basement with the corrected gravity and magnetic data, and they drew the first-class basement geological map of the southwest basin. Chakravarthi et al. [26] designed a comprehensive geophysical method including the deep resistivity method and gravity data to determine the thickness of the sedimentary basin. Eirik et al. [27] applied a high-resolution seismic reflection method to the mapping of Quaternary strata in the Tana delta of northern Norway. Xavier et al. [28,29] successively used the resistivity method and GPR to study the internal structure characteristics of Quaternary sediments in central Ireland, and they used time-lapse resistivity imaging method to analyze Quaternary sediments. Lyons et al. [30] used drilling exposure and seismic reflection data to divide the late Quaternary strata of the Lake Malawi rift in East Africa. Yang et al. [31] used magnetotelluric methods to reveal the distribution and thickness of the Cenozoic sedimentary layers in the Gonghe Basin.

**Figure 1:**
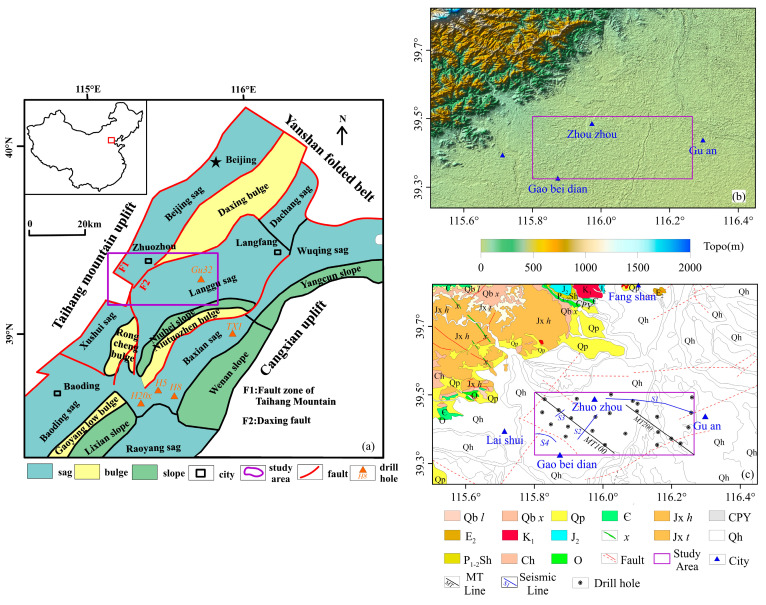
Background of the study area. (**a**) Structural background of the Jizhong Depression (modified from [35]). (**b**) DEM of the study area. (**c**) Geological map of the study area. (The geological map is sourced from the China Geological Survey, Geological Cloud Platform, Basic Geological Database.)

A digital elevation model (DEM) (Aster GDEM, http://datamirror.csdb.cn/admin/datademMain.jsp, accessed on 15 July 2024) reveals the landscape structural characteristics of the North China Plain and its adjacent areas (Figure 1b). The study area is located in front of Taihang Mount in the North China Plain. The terrain here is flat (Figure 1b), and the accumulation of alluvial deposits is extremely thick (Figure 1c). Except for the expensive drilling, it is difficult to carry out the geological survey the traditional way. Therefore, geophysicists and geologists should work under the same geological target [32], 1998) in order to apply geophysical methods to a modern geological survey with a thick coverage area to improve the mapping efficiency and infer the geological structure under overburden [33]. However, the combination of these geophysical techniques has not yet been used as a mapping tool in the geological survey in the thick overburden area of the North China Plain. The China Geological Survey (CGS) set up a pilot project for mapping special geological and geomorphic areas, aiming to explore and innovate mapping technology and methods, and reveal the resource status of these areas and the major scientific problems covered [34]. In order to explore the application of geophysical technique in geological mapping, we selected a site in Zhuoxian, which is located in the northwest of the North China Plain, to carry out our research. The site contains the geomorphic characteristics of a piedmont alluvial proluvial plain, showing various sedimentary and lithologic environments. In the process of geological mapping in the survey area, we utilized the results of geophysical surveys and traditional surveying techniques (drilling) to reconstruct the basement structural framework and stratigraphic structure of the area through a fine inversion interpretation of the collected geophysical dataset, providing an important basis for the tectonic movement and sedimentary environment evolution of the area.

## 2. Geological and Geophysical Setting

### 2.1. Geological Background

The Jizhong Depression is located in the western part of the Bohai Bay Basin and is a Mesozoic Cenozoic sedimentary depression developed on the basement of the North China ancient platform. There are four uplifts around it: the Yanshan uplift in the north, the Xingheng uplift in the south, the Taihang uplift in the west, and the Cangxian uplift in the east. The research area is located in the central part of the Jizhong Depression (Figure 1a), covering the main structural units of the Langgu Sag, Daxing uplift, Beijing seg, and Taihang Mount structural belt. These structural units correspond to high gravity anomalies and low gravity anomalies, and their boundaries are controlled by faults that cut into the bedrock [36]. Some scholars have carried out a detailed division and correlation of pre-Cenozoic strata in the depression [37,38,39,40,41]. On this basis, He et al. [35] established a comprehensive stratigraphic histogram of the Jizhong Depression by using the latest drilling and seismic data.

### 2.2. The Physical Properties of Stratum

The physical properties of sediments and bedrock are essential to determine the geophysical methods used and subsequent geological interpretation. At the beginning of the work, the physical properties and logging data of the study area and its adjacent areas were collected.

**Figure 2:**
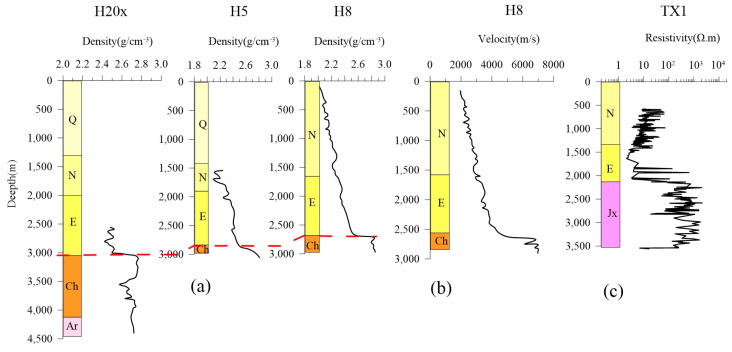
Borehole logging curve of the Jizhong Depression. (**a**) Density logging curve [42]. (**b**) Velocity logging curve [42]. (**c**) Resistivity logging curve.

The geological structural units in the Jizhong Depression have experienced the same geological process. Therefore, the physical characteristics of the strata are consistent. We collected density, acoustic, and resistivity logging data from several boreholes (see Figure 1a for well locations) in the adjacent study area as physical property references to study the physical properties of underground media. The density and wave velocity data were from the North China Plain oil logging [42], and the resistivity data were from the geothermal borehole logging in the new area of Xiongan. From Figure 2a, it can be seen that the density of the strata increases with the depth; that is, the density and depth of the strata basically have a positive correlation. The density difference between the Cenozoic strata and the underlying strata between different boreholes is significant and relatively stable, which is the main density interface. The variation in wave velocity has a similar morphology to the density of the strata (Figure 2b). The resistivity characteristics are relatively complex. From Figure 2c, it can be seen that the resistivity in the Cenozoic is low (especially in E layer). After entering the Paleozoic, the resistivity suddenly increases by two orders of magnitude, which is characterized by high resistivity. It is worth noting that the resistivity in the Cenozoic decreases with depth, which may be related to the high shale content in the Tertiary strata. The logging results show significant differences in density, wave velocity, and resistivity between the Quaternary and Tertiary, as well as the Tertiary and Paleozoic interfaces, providing a good prerequisite for geophysical exploration.

## 3. Data

The differences in density, wave velocity, and resistivity among different strata in the research area are prerequisites for conducting geophysical exploration. We used gravity, electromagnetic methods, and reflection seismic methods to study the sedimentary morphology, fault distribution, and fine structures of the Cenozoic in the uplift area. In order to economically and effectively obtain the basic structural framework and detect depth changes on the bedrock surface, 1:50,000 gravity measurements were conducted throughout the entire study area. Meanwhile, we conducted magnetotelluric (MT) measurements on two profiles to depict the deep subsurface sedimentary structure. To further determine the fault attributes delineated by gravity and obtain the structural style of the bedrock uplift area, we deployed four shallow seismic profiles. The geophysical exploration deployment is shown in Figure 1c.

### 3.1. Gravity

**Figure 3:**
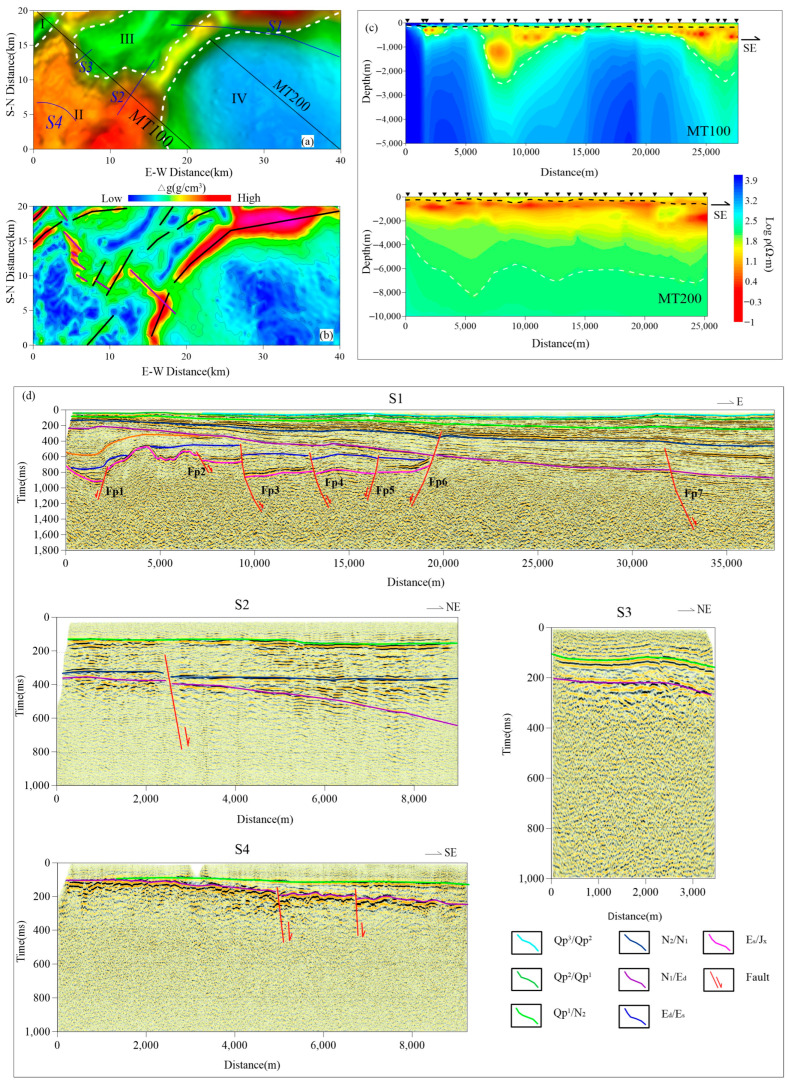
Comprehensive geophysical interpretation map. (**a**) Bouguer gravity anomaly map. (**b**) Bouguer gravity horizontal gradient model. (**c**) Two-dimensional (2D) inversion resistivity profiles of MT100 and MT200. (**d**) S1, S2, S3, S4 are reflection seismic time profiles.

The ground gravity is carried out in a grid of 500 m by 500 m. First, we obtained the Bouguer gravity anomaly (BGA), as shown in Figure 3a. It can be seen that the BGA reflects the structural framework of high in the west and low in the east, which is consistent with the trend of the regional geological structure. According to the abnormal distribution, the study area can be divided into four abnormal areas from west to east, namely, the low abnormal area in the west (I), the low abnormal area in the north (III), the low abnormal area in the southeast (IV), and the wide and gentle high abnormal area (II). Compared with the geological structure map (Figure 1a), it was found that they correspond to the Taihang Mountain uplift, Beijing Sag, Langgu Sag, Daxing Bulge (northeast branch), and the Taihang Mount structural belt, respectively.

As a whole, the anomaly is high in the west and low in the east, reflecting the change in sedimentary thickness, which may be the result of the uplift of the mountains in the northwest and the faulting of the eastern basin. In addition to the high anomaly in the northwest caused by the shallow depth of bedrock, the anomaly in the east is relatively low, and the anomaly increases from east to west, which may mean that the Cenozoic overburden in the east is thicker than in the other parts. From the perspective of zoning, the BGA of zone IV (Langgu Sag) is the lowest, indicating that the thickness of the sedimentary layer in the southeast is large. Judging from the abnormal amplitude, the sedimentary center may be located in the southeast. The BGA of zone II (Taihang Mont structural belt) is abnormally high, indicating thin deposition and shallow bedrock. The abnormal value of zone III (Beijing Sag) is relatively low but higher than that of zone IV (Langgu Sag), indicating that there is a certain thickness of deposition, but the sedimentary layer is relatively thin and the bedrock is relatively shallow. Zone I has the lowest value in the overall high background, considering it is a small sag between Taihang Mountains. By calculating the horizontal gradient model of the Bouguer gravity anomaly, we have obtained the plane distribution of the faults in the study area. From Figure 3b, it can be seen that the boundaries of the four zones are very clear, with the largest boundaries in the northeast and northeast of the Langgu Sag and Daxing uplift, representing the main structural direction of the study area (black line in Figure 3b). There are a series of secondary faults along the direction of the main fault, which are influenced by the northeast structure and take the form of multi-level faults (purple line in Figure 3b). In order to study the fault style of the abnormal zoning and the bedrock characteristics corresponding to the abnormal high value area, seismic section S1 is deployed across the second, third and fourth abnormal zoning, and seismic sections S2 and S3 are deployed between the second and third abnormal zones to identify the unconfirmed Songlindian fault [43] (purple line in Figure 3b), and seismic section S4 is deployed in the southwest of zone II to obtain the accurate bedrock depth in the area.

### 3.2. Magnetotelluric

In this work, the magnetotelluric method was used to detect basement relief and deep geological structures. Four component data (Hx, Hy, Ex and Ey) were collected along two sections (MT100, MT200). A total of 65 magnetotelluric stations were deployed with an average station spacing of 2000 m. A multifunctional electromagnetic method system [44] running for at least 300 min at each station was used. In data processing, frequency spectrum analysis and digital filtering were used to eliminate 50 Hz interference and other high-frequency background noise; tensor impedance elements were used to suppress non-Gaussian noise interference [45,46]. MT data qualitative analysis was carried out by tensor impedance, and impedance tensor decomposition was carried out to analyze the structural dimension, principal axis azimuth and polarization pattern of the study area [47]. The correction of apparent resistivity and impedance phase included static effect correction by the median filtering method [48]. Firstly, the Bostick imaging results of each measuring point were used as the initial model of one-dimensional inversion [49]. Based on the comparison and analysis of geological data and one-dimensional inversion results, a two-dimensional initial geoelectric model was constructed. The joint inversion of TE and TM polarization modes was carried out by two-dimensional nonlinear conjugate gradient (NLCG) inversion [50]. The inversion results are shown in Figure 3c.

Section MT100 passes through the west side of the study area along the NW direction. The inversion resistivity section (Figure 3c) reveals the “two sags and one bulge” structural framework, which corresponds to four divisions of BGA, respectively. The whole resistivity profile has the characteristics of high–low–high resistivity, and its interface is clear (black blue dotted line). The underground multi-layer geoelectric properties can be interpreted as Quaternary sediments, Tertiary sediments and basement. From the MT inversion map, it can be seen that the Quaternary sediments in the four divisions of BGA are thickened from west to east, ranging from dozens to 300 m. The west section of the survey line is close to the exposed area of bedrock, and the overburden almost disappears, which is characterized as high overall. The resistivity value (about 5000 Ω·m) is significantly higher than that of the overburden of non-foundation rock. The Tertiary sediments are mainly accumulated in the sag, of which zone I is about 500 m, zone III is about 2200 m, zone IV is about 2000 m, the bedrock buried in the bulge area is relatively shallow, only a dozen meters near the northwest side of the Piedmont, and the shallowest part in the northwest end of zone II (Taihang Piedmont structural belt) is about 200 m. Different resistivity blocks below the Tertiary sedimentary layer may correspond to different strata or intrusive rocks.

The MT200 line is also laid in the northwest direction. The northwest end of the inversion resistivity section (Figure 3c) is located at the boundary between BGA II and IV, mainly crossing BGA low anomaly IV. The MT resistivity inversion section as a whole presents high–low–high electrical characteristics, with clear interface, corresponding to Quaternary, Tertiary deposits and basement, respectively. The line MT200 is basically located in the low abnormal area of BGA Area IV, which corresponds to the main part of the Langgu Sag. The Quaternary and Tertiary system deposits are very thick, and the bedrock is deep. The northwest side of the survey line is close to the Daxing Bulge, which is the transition area between the Daxing Bulge and Langgu sag. Affected by the Daxing Bulge, the overburden is slightly shallow, and the bedrock buried depth is about 3000 m. The buried depth of bedrock varies from 5500 m to 8500 m in the whole sag, and the thickness of Quaternary and Tertiary sedimentary layers changes gently on the whole, indicating that the sedimentary rate changes slightly along the structural strike direction, and the sedimentation is relatively stable. In the whole section, the geoelectric characteristics are in good agreement with the regional structure and sedimentary model.

### 3.3. Reflection Seismic

**Figure 4:**
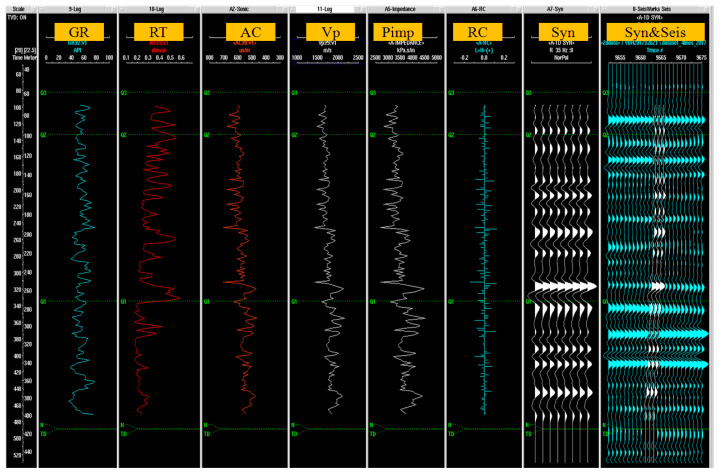
QGJ01 borehole logging curve and geological horizon calibration.

The seismic data acquisition used Sercel 428XL. The instrument recorded data from 2000 channels. The P-wave receiver adopts the EST-20dx series 60 Hz detector string. The excitation source uses the T15000 vibrator produced by IVI company for excitation with a 6000 pound force output and a broadband 10–550 Hz high-frequency output. In our study, the main detection targets of shallow seismic are the middle and shallow structures of sedimentary layers, fault attributes and BGA highly abnormal basement morphology. A total of four shallow seismic profiles are deployed, including the NE strike S2, S3 and WE strike S1 and S4 profiles (Figure 3c), which are more than 40 km in total. It mainly covers the key areas, especially the boundary between the bulge and the sag, as well as the speculated Songlindian fault (Figure 1c), which can provide basic data for the inversion of the bedrock surface and regional stability evaluation. In order to obtain accurate formation wave velocity, in seismic data processing, we use acoustic logging data and seismic wavelet convolution to generate synthetic seismic records [51], and then we compare them with seismic channels near the well. The seismic reflection layer is precisely calibrated by geological layers. Figure 4 is the QGJ01 borehole logging curve and geological horizon calibration map. Through processing and inversion, we have obtained the time section and interpreted the reflected wave group on each section according to the wave group strength, continuity and geological data.

The S1 profile shows seven sets of reflected wave impedance interfaces, among which the main interfaces are the Quaternary bottom interface Qp1, the Neogene top and bottom interfaces N2 and N1, and the Paleogene Dongying Formation and Shahejie Formation interfaces Ed and Es. The discontinuous wave impedance interface corresponds to seven sets of faults, and none of the faults have penetrated into the fourth proximal strata. The left side of fault Fp1 is a stratigraphic depression area, corresponding to the BGA II area. On the right side of the fault is a stratigraphic uplift area, corresponding to the BGA I zone. Based on comprehensive regional geological analysis, this fault is the southern extension of the Nanyuan fault in Tongxian County. The depression area on the left side of the fault corresponds to the Beijing Depression, and the bulge area on the right side of the fault corresponds to the Daxing Bulge. Fp2, Fp3, Fp4, Fp5, Fp6, and Fp7 belong to the branch faults of the Daxing fault zone. Based on the characteristics of the fault, it is inferred that the Fp2 fault is the main fault. The Daxing uplift is located on the left side of the fault zone, and the strata on the right side of the fault zone gradually sink into the Langgu Sag.

Three sets of reflected waves were interpreted on the S2 profile, suggesting that they are reflections from the Quaternary bottom interface, Neogene interface, and Paleogene interface. A set of faults was inferred and explained on this profile with fault segment N1 at the bottom boundary. It is worth noting that there is a corresponding relationship between the projection of the fault on the ground and the inferred location of the Songlindian fault based on gravity exploration results. On the S3 section, two groups of reflected waves are relatively clear, and it is inferred that they are reflected by the Quaternary bottom boundary and Neogene bottom boundary.

On the S4 section, two sets of reflected wave phases are inferred to be reflections from the Quaternary and Neogene bottom boundaries. The two sets of wave resistance interfaces exhibit characteristics of shallow western and deep eastern layers. The western section has thin sedimentary layers and shallow bedrock, while the eastern section has thick sediments and deep bedrock. Two sets of faults were interpreted at the discontinuity of wave impedance, and the fault did not cross the Quaternary strata.

In general, the bedrock interface in the study area fluctuates greatly, showing the characteristics of high in the west and low in the east. In the detection area of S2, S3 and S4 survey lines in Zhuoxian County in the west of the survey area, the shallowest buried depth of bedrock is less than 200 m, while in the Langgu Sag in the east of the survey area, the buried depth of bedrock can reach several kilometers (no bedrock is detected in the sag area). In the Daxing Bulge area where the S1 survey line passes, the bedrock buried depth is about 1000 m. The spatial distribution of the Daxing Fault has been finely depicted, and the fault horizon has been determined, which provides a basis for the evaluation of fault activity. The fault obtained from the S2 profile combined with gravity exploration results confirmed the existence and nature of the Songlindian fault.

### 3.4. Borehole

**Figure 5:**
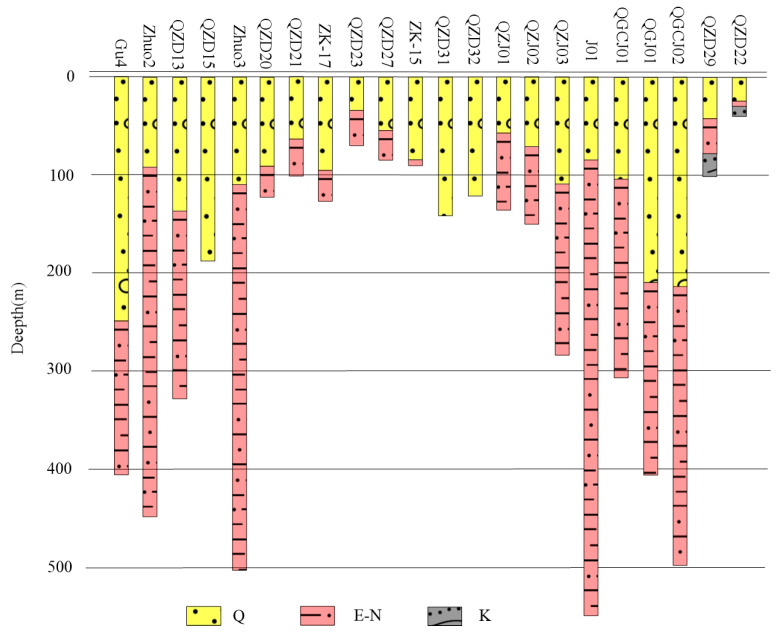
Geological drilling stratum map of the survey area.

Drilling is the most powerful tool for directly and accurately revealing the thickness and lithology of sedimentary layers. The eastern part of the research area (BGA low value anomaly area) is located in the Langgu Sag, which is a favorable area for oil exploration. Therefore, there is a lot of drilling work in this area, and the deep geological information in this area is complete. However, the western part of the research area is close to the uplift zone, where there is little geological drilling work and basically a blank space. The drilling data mainly come from this research work. These boreholes are evenly distributed throughout the study area, some of which were obtained from previous hydrogeological work. Its location distribution is shown in Figure 1c. The depth of the geological interface is shown in Figure 5.

## 4. Methodology and Result

### 4.1. Implementation Method

The main purpose of this article is to establish a structural framework for the survey area based on geophysical data, expose sedimentary layers, and obtain the bedrock interface (the bottom interface of the Tertiary). Due to the fact that the density of sedimentary layers in a basin is often lower than that of bedrock, significant low gravity anomalies can be observed on sedimentary basins, which can be used to invert the basement characteristics of the basin. Usually, the method of using gravity data to invert the basement assumes that the Quaternary and Tertiary sedimentary layers are uniform [52,53,54,55], or the density of the Tertiary strata is uniform. In fact, the density of the Quaternary period increases with depth [56,57,58,59]. Therefore, this assumption of neglecting local anisotropy can only achieve satisfactory results when the geological structure is simple or the study area is large enough [60,61,62]. The area of this study is about 800 km^2^, and the inhomogeneity of underground media cannot be ignored. The difference between drilling and rock density also confirms this fact.

In view of this problem, we have integrated geological, drilling and various geophysical data, and adopted the following strategies. First of all, we make full use of the drilling information and geophysical profile results of inversion under the constraint of drilling to obtain the main formation interface depth under the control of drilling and geophysical profiles. Then, combined with the statistical data of density logging and core density measurement in the survey area, we establish a three-dimensional density difference model of the survey area. Finally, we use the 3D variable density gravity inversion method to obtain the bedrock depth in the study area bedrock interface [63].

### 4.2. Establishment of Density Difference Model

The underground strata exhibit non-uniformity in both horizontal and vertical directions. Therefore, density difference models cannot be constructed using constants. The density statistical parameters of the survey area (Table 1) were obtained from density logging and core density testing of oil boreholes in the southeastern part of the Jizhong Depression [42]. In the study of density variation with depth, drilling data are the most effective, but only one deep oil well (Gu32) that penetrated the Cenozoic era was collected in the survey area. Therefore, in the process of establishing the density model, the acquisition of the depth of the geological interface is mainly based on two magnetotelluric profiles and four seismic profiles in the survey area (Figure 1c).

Based on the characteristics of the cross-section and the needs of regional control, a total of 25 control points were selected for the study. In the same coordinate system, we accurately pick the coordinates of each profile control point and the depth of the interface layer on the plane, and we use the exponential density difference depth function to establish a density difference model, as shown in Formula (1):

(1) $$\Delta_{\rho }(z)=\Delta \rho_{0}e^{-\lambda z}$$

**Figure 6:**
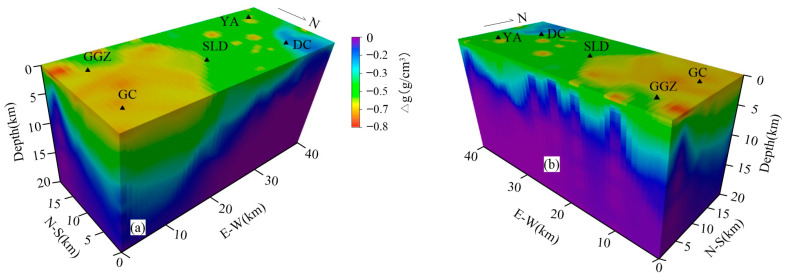
Three-dimensional (3D) density difference model of study area. (**a**) Northeast perspective. (**b**) Southeast perspective. Abbreviations: Songlindian (SLD), Gongcun (GC), Yian (YA), Gaoguan-zhuang (GGZ), Dongchengfang (DC).

Among them, $\Delta_{\rho }$ is the density difference, $\Delta \rho_{0}$ is the surface density difference, z is the depth, and λ is the density changes with the depth factor. The least square fitting method is used to obtain the $\Delta \rho_{0}$ and λ, and then the data grid is used to obtain the sum of the whole study area’s λ and $\Delta \rho_{0}$. Using the combination of the sum of the points, we obtain the 3D variable density model of the study area (Figure 6).

### 4.3. Interface Estimation of Sedimentary Depth

**Figure 7:**
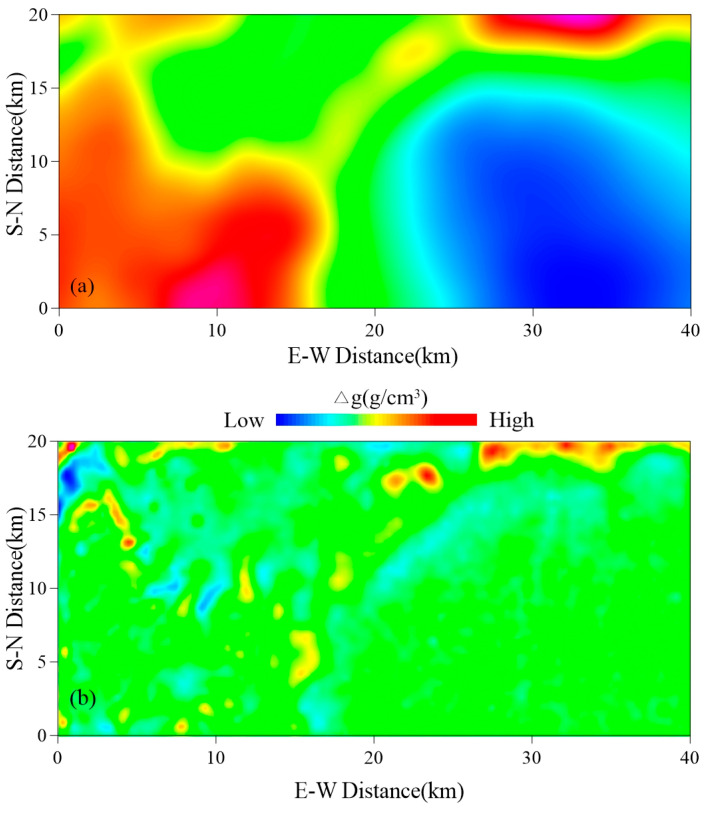
(**a**) Regional gravity anomaly. (**b**) Residual Bouguer gravity anomaly.

Bouguer gravity anomaly is a comprehensive reflection of all density inhomogeneous bodies in the ground, which can be divided into residual gravity anomaly and regional gravity anomaly. Generally speaking, the residual gravity anomaly reflects the characteristics of the shallow density inhomogeneous body (such as the sag and bulge of the basin basement), while the regional gravity anomaly reflects the structural characteristics of the deeper layer. Using the method of multiple iteration slip trend analysis [64], different window sizes and trend surface orders are selected for calculation. Then, the regional field is separated (Figure 7a). After comparison with the basin structure revealed by the comprehensive geophysical section, the residual Bouguer gravity anomaly, which can better reflect the basin structure in the survey area, is obtained (Figure 7b).

As mentioned earlier, MT inversion profiles and seismic profiles reveal the undulations of the Tertiary and basement interfaces. Furthermore, based on the interface exposed by drilling and the density values of the main strata, we constructed a 3D variable density model of the entire study area and used the variable density iteration method to invert the bedrock interface [52]:

(2) $$\begin{array}{l} p^{\left(k\right)}(x_{i}+y_{i})=p^{\left(k-1\right)}(x_{i}+y_{i})\\ +\frac{g^{obs}(x_{i}+y_{i})-g^{cal}(x_{i}+y_{i})}{2\pi G\Delta \rho (x_{i},y_{i},z)},i=1,\ldots ,N \end{array}$$

where G is the constant of universal gravitation; $p^{\left(k\right)}(x_{i}+y_{i})$ and $p^{\left(k-1\right)}(x_{i}+y_{i})$ are the ith inversion results of the k and k+1 calculation points, respectively; $\Delta \rho (x_{i},y_{i},z)$ is the density difference at the ith point, whose changes along the depth z can be expressed in the form of Formula (1); $g^{obs}(x_{i}+y_{i})$ and $g^{cal}(x_{i}+y_{i})$ are the measured gravity anomaly at the ith point and the forward gravity anomaly at the second iteration, respectively. When calculating the gravity anomaly, the sedimentary layer above the basin basement is divided into vertically juxtaposed prisms, which are directly below the gravity calculation point, the number of prisms is the same as the calculation point, and the horizontal dimension is the same as the calculation point spacing. The iteration is terminated when the $g^{obs}(x_{i}+y_{i})$ and $g^{cal}(x_{i}+y_{i})$ mean square deviation of sum is less than the noise of gravity data.

**Figure 8:**
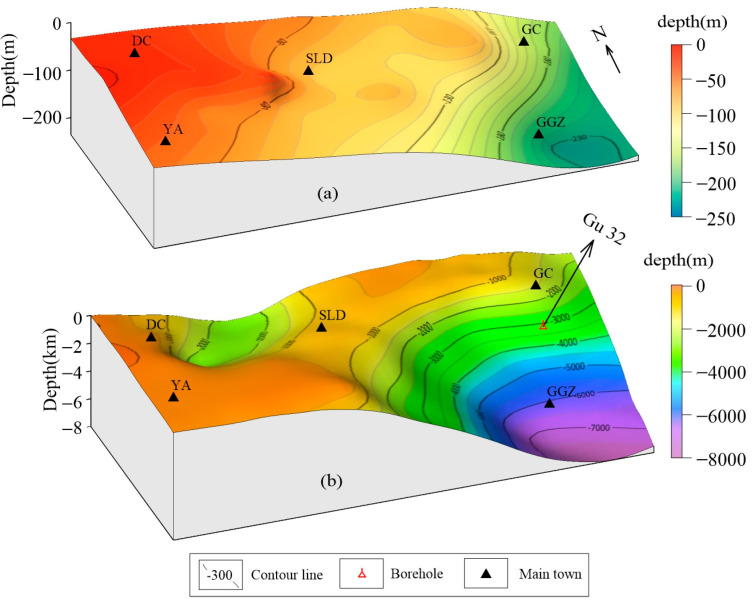
(**a**) Quaternary bottom interface. (**b**) Tertiary bottom interface. The place name is consistent with Figure 7.

By comparing the inversion depth value of the Paleogene bottom boundary with the actual value of the Gu32 hole (Figure 8b), the inversion depth of the Paleogene bottom interface at Gu32 (Location as shown in Figure 1a) is 3151.84 m, the exposed depth of borehole is 3092.37 m, and the error is 1.92%. The inversion result is verified by borehole.

Thanks to the Quaternary geological survey, shallow geological drilling in the study area almost penetrated the Quaternary, and important areas are controlled by geophysical profiles. Therefore, by combining drilling with the interface depth points obtained from geophysical profiles, we can more accurately obtain the Quaternary bottom boundary (Figure 8a).

The western part of the study area is the bulge area and the eastern part is the sag area. In general, the eastern sag has a large sedimentary thickness. The inversion results clearly show the characteristics of the Langgu Sag. The main depth of the sag basement is 3–8 km. From the perspective of basement morphology, the sag center is southeast. From the depth change in Tertiary sediments, the thickness in the southeast is large, up to 8000 m, while the thickness in the northwest is generally less than 200 m, and the thickness in the north and west is significantly less than that in the east. From the change in sedimentary thickness, the Beijing Sag in the northwest has been filled by the alluvial proluvial materials of the rivers in front of Taihang Mount (mainly Juma river) since the Paleogene, and it has been basically filled up by the Quaternary. Meanwhile, the eastern part of the Langgu Sag is filled with alluvial deposits from the west (Juma River) and north (Yongding River). The thickness of the Quaternary system of the Juma River alluvial fan in the western survey area is generally 30–120 m, and it is thick in the east and thin in the west. It is controlled by the basement structure and slopes gently towards the east. The thickness of the Quaternary system in the Yongding River alluvial fan in the eastern part of the survey area is generally 120–240 m. Sediments are thin in the convex area and thick in the bulge area. The thickness of the Daxing Bulge is about 150–180 m, and the thickness of the Langgu Sag is about 220–240 m.

## 5. Discussion

Through the comprehensive geophysical method and geological drilling, we have obtained the bathymetric map of the Quaternary and Tertiary bottom interfaces in the whole survey area. The petroleum drilling in the Jizhong Depression shows that the sedimentary strata in this area are Quaternary, Neogene, Paleogene, great wall system and Jixian system from top to bottom, and the Mesozoic and Paleozoic strata are missing, which indicates that this area is a denuded area at least before the Paleogene. According to the analysis of apatite/zircon fission track results by Meng et al. [65], the uplift of Taihang Mount in the Cenozoic is unbalanced, and it experienced uplift in the early Paleogene. The rapid uplift in the Neogene shaped the main body of Taihang Mount (23–18 Ma) with an uplift range of 4000 m. Although there are different opinions on the stage and clear time of the event, most studies have concluded that a strong uplift occurred at about 23 Ma [66,67,68]. The Paleocurrent direction of north Jumahe River in the study area is east–west, and the southeast of the area is the main sedimentary area in this period. In the Oligocene, the Taihang Mount rose rapidly, and the eastern plain basin began to rift. The Dongying Formation and Guantao Formation of the Paleogene are widely developed in this area, which are Oligocene to Miocene strata. The Dongying Formation is thin in the front of the mountain in the northwest and thick in the southeast, up to 1000 m. The NE–NNE trending normal faults in the study area developed with the uplift of Taihang Mount. The western part of the Jizhong Depression (including the study area) was in a high structural position, resulting in a large number of denudation of the strata [69]. Since the late Pleistocene, the study area has continued to experience strong tectonic movement. A series of alluvial fans are developed in the front of the western mountain, the thickness of which is equivalent to that of the Quaternary, and it generally increased from west to east, reflecting the migration direction of the Quaternary alluvial fans. This geological survey and remote sensing image have reached similar conclusions. The western mountain area will continue to rise in the future. The results of this comprehensive geophysical exploration are highly consistent with this geological understanding.

## 6. Conclusions

In this study, we use comprehensive geophysical data from different sources, combined with geological and borehole data, to detect basement relief and fault distribution. Using geophysical data and borehole data, the geological framework of the study area and the thickness changes of the strata under some section points are revealed. Under the constraints of MT, seismic and borehole, the density contrast model is established. Finally, the fluctuation pattern of the Cenozoic bottom interface is obtained by gravity data inversion.

There is a great difference in the sedimentary thickness between the western area and the eastern area. The shallowest part of the western is about 80 m, and the deep part of the eastern area is up to 8000 m. The change in the sedimentary thickness in the eastern and western areas is nearly 7000 m, which is caused by the uplift of the surrounding mountains. The NE trending fault dominated by the Taihangshan fault controls the sedimentary formation of the basin in the study area, which is confirmed by the depth interface map of the Tertiary and Quaternary system (Figure 8). These results are in good agreement with the regional and local geological background. Using the comprehensive geophysical data, we obtain the relief surface of the bedrock, the thickness of the sedimentary layer and the structural style of the important convex area, which provide a new perspective for the study of the geological structural evolution. Different geophysical methods have different characteristics and advantages. Magnetotelluric and seismic survey can provide high-precision and deep underground geological structure information, but considering the cost and work efficiency, it is almost impossible to implement in a large area. Gravity survey can make up for this deficiency, but it lacks depth resolution. Therefore, in the geological survey of thick coverage area, both efficiency and success are considered under this premise, how to optimize the comprehensive geophysical method is an important problem worthy of further study.

Although we have obtained the Quaternary and Tertiary bottom interfaces through comprehensive geophysical methods combined with geological data, we do not yet have a good understanding of the 3D geological structure and formation lithology of the study area. The sedimentary environment of the study area is complex and affected by many factors. The results of the study can establish a regional structural framework and stratigraphic structure as a whole, but they lack sufficient details. In order to provide detailed support for geological survey, the application of shallow geophysical methods should be carried out according to the needs of hydrology and environmental geology.

## Figures and Tables

**Table 1:** Statistical parameters of density.

Age		$\mathrm{Density}/(g\cdot {\mathrm{cm}}^{-3}$)	
		Range	Average
Quaternary	Q	1.133–2.332	1.94
Neogene	Ng	2.237–2.283	2.25
Paleogene	Ed-Es_1_	2.220–2.350	2.31
	Es_2_-Es_3_	2.250–2.500	2.38
	Es_4_-Ek	2.330–2.550	2.44
Proterozoic	Jxw, Chg, Pt	2.610–2.800	2.66

## Data Availability

Data associated with this research are available and can be obtained by contacting the corresponding author.
